# Predictive modeling and machine learning show poor performance of clinical, morphological, and hemodynamic parameters for small intracranial aneurysm rupture

**DOI:** 10.1038/s41598-025-08478-1

**Published:** 2025-07-05

**Authors:** Vanessa M. Swiatek, Samuel Voss, Florian Sprenger, Igor Fischer, Hafez Kader, Klaus-Peter Stein, Roland Schwab, Sylvia Saalfeld, Ali Rashidi, Daniel Behme, Philipp Berg, I. Erol Sandalcioglu, Belal Neyazi

**Affiliations:** 1 Department of Neurosurgery, Otto-von-Guericke University, Magdeburg, Saxony Anhalt Germany; 2 Department of Fluid Dynamics and Technical Flows, Otto-von-Guericke University, Magdeburg, Saxony Anhal Germany; 3 Department of Medical Engineering, Otto-von-Guericke University, Magdeburg, Saxony Anhalt Germany; 4 Forschungscampus STIMULATE, Magdeburg, Saxony Anhalt Germany; 5 Department of Medical Informatics, University Hospital Schleswig-Holstein Campus Kiel, Kiel, Schleswig-Holstein Germany; 6 Department of Neuroradiology, Otto-von-Guericke University, Magdeburg, Saxony Anhalt Germany; 7 Department of Neurosurgery, University Hospital Düsseldorf and Heinrich-Heine-University, Dusseldorf, North Rhine-Westphalia Germany; 8 Autonomous Multisensor Systems Group, Institute for Intelligent Cooperating Systems, Otto-von-Guericke University, Magdeburg, Saxony Anhalt Germany

**Keywords:** Small intracranial aneurysms, Rupture risk assessment, Semiautomatic neck curve reconstruction, Computational fluid dynamics, Predictive modeling, Neuro-vascular interactions, Outcomes research, Stroke

## Abstract

Small intracranial aneurysms (SIAs) (< 5 mm) are increasingly detected due to advanced imaging, but predicting rupture risk remains challenging. Rupture, though rare, can cause devastating subarachnoid hemorrhage. This study analyzed 141 SIAs (101 unruptured, 40 ruptured) using semi-automatic morphological analysis and high-resolution, image-based blood flow simulations from 3D rotational angiography. Advanced morphological and hemodynamic parameters were extracted, with clustering applied to address multicollinearity. Univariate logistic regression identified cluster representatives, and forward selection highlighted the maximum height, Neck inflow rate, and Non-sphericity index as rupture predictors, though only the latter two were significant. Clinical variables like age, sex, and comorbidities were also assessed but failed to predict rupture risk. The full model showed overfitting, with a pseudo-R^2^ of 0.142 on the training set but only 0.032 on the test set. A simplified model using just Neck inflow rate and Non-sphericity index performed similarly poorly (pseudo-R^2^ of 0.034). Multiple machine learning classifiers were evaluated, with similar performance across models, supporting the model-independence of the results. Overall, neither morphological, hemodynamic, nor clinical variables reliably predicted rupture risk, highlighting the limitations of current methods and underscoring the need for prospective studies and multimodal approaches that integrate imaging biomarkers and compare small and large aneurysms for better risk stratification.

## Introduction

The widespread availability of advanced neuroimaging has significantly increased the detection of unruptured intracranial aneurysms (IAs), including small, asymptomatic aneurysms^1,2^ with a diameter of less than 5 mm. They have an estimated prevalence of approximately 3–5% in the general population^3,4^encompassing both ruptured and unruptured cases. The rupture of an aneurysm can result in devastating subarachnoid hemorrhage (SAH), with morbidity and mortality rates reaching 25% and 40%, respectively^4^. These clinical challenges highlight the need for precise rupture risk stratification, balancing the potential benefits of intervention against the procedural risks associated with microsurgical clipping or endovascular treatment^5^.

While aneurysm size has traditionally been regarded as a primary determinant of rupture risk^6,7^ discrepancies between observational studies and clinical findings challenge this view. Small aneurysms frequently appear in ruptured cases^8–12^ suggesting that size alone is insufficient to predict rupture. Additionally, morphological features offered a clearer indication of aneurysm disease status in some studies, with shape-related parameters—particularly those reflecting irregularity—emerging as more reliable predictors than size alone^13^. This has driven the exploration of additional factors, including clinical, morphological, and hemodynamic parameters, to refine risk assessment. Several risk factors for the rupture of small IAs (SIAs), defined as those with a diameter ranging from 5 to 7 mm according to different studies, have been identified. These include younger age (< 50 years)^8,14^ aneurysm diameter ≥ 4.0mm^8^, hypertension^8,12,14^ and the presence of multiple aneurysms^8^. A recently published meta-analysis by Pettersson et al., focusing on SIAs smaller than 7 mm, also highlighted patient age as a significant predictor in univariate analysis^15^. However, through multivariable analysis, the study identified four morphological parameters (size ratio^16,17^ aspect ratio^16^ bifurcation point, and aneurysm irregularity) and two hemodynamic parameters (pressure loss coefficient and wall shear stress (WSS)) as independent predictors of rupture^15^. Pettersson et al. concluded that morphology-related predictors outperform traditional demographic predictors commonly used in scoring systems. These findings underscore the critical role of aneurysm morphology in rupture risk assessment^15^.

Accurate morphological evaluation is further important because it directly impacts the reliability of hemodynamic simulations, which are essential for understanding aneurysm pathophysiology^17–22^. Hemodynamic forces play a pivotal role in the initiation, growth, and rupture of aneurysms^23–25^. Image-based blood flow simulations based on computational fluid dynamics (CFD) studies have highlighted WSS, the tangential force of blood flow on vessel walls, as a key factor driving aneurysm progression^26–30^. Emerging evidence supports a dual-pathway hypothesis: low WSS combined with a high oscillatory shear index induces inflammatory-cell-mediated remodeling, typically associated with larger, atherosclerotic aneurysms, whereas high WSS and a positive WSS gradient trigger mural-cell-mediated remodeling, more often linked to smaller, thin-walled aneurysms^31^. This hemodynamic heterogeneity underscores the need for comprehensive evaluation, integrating clinical, morphological, and hemodynamic data for a more accurate rupture risk assessment.

This study builds on these findings, focusing on SIAs (with an aneurysm size < 5 mm as defined by the SUAVe study^8^) and their rupture risk. By integrating clinical, morphological, and hemodynamic data, we aim to refine our understanding of the risk factors driving rupture in SIAs.

## Materials and methods

For this study, we analyzed a retrospective database comprising 300 patients with 512 IAs treated at the Department of Neurosurgery, Otto-von-Guericke University Hospital, Magdeburg, Germany, between 2006 and 2020. Each patient in the database had undergone 3D rotational angiography, which allowed for the semi-automatic reconstruction and segmentation of individual 3D surface models of the aneurysms and their parent vessels. The cohort was refined based on the following inclusion criteria:

1. Aneurysm size < 5 mm.
2. Compatibility of the 3D surface model for semi-automatic reconstruction of the SIA morphological features and for hemodynamic analysis using CFD.

**Fig. 1:**
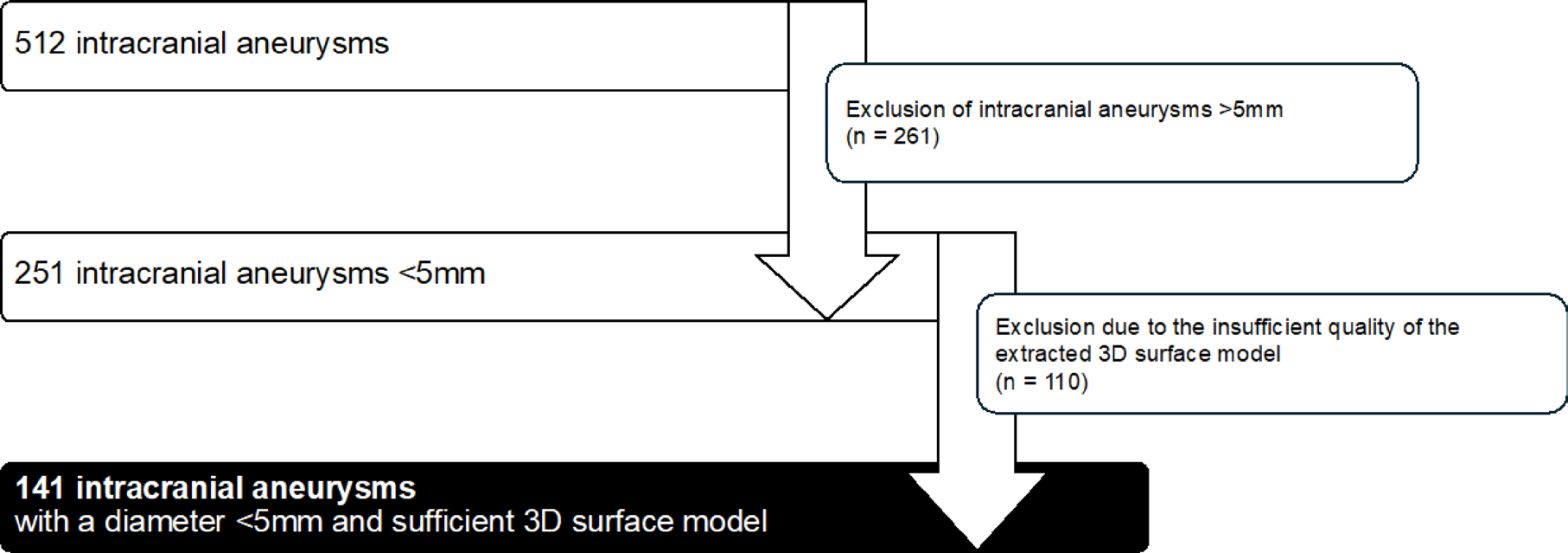
Flowchart illustrating the selection process for SIAs included in the study. Out of 512 IAs identified in 300 patients, 261 were excluded due to a diameter > 5 mm, and 110 were excluded due to insufficient quality of the extracted 3D surface model. The final analysis included 141 SIAs with a diameter < 5 mm and sufficient 3D surface model quality.

In accordance with the inclusion criteria, 261 IAs with a diameter > 5 mm and 110 SIAs with insufficient quality in the extracted 3D surface models were excluded, leaving a total of 141 SIAs for analysis (Fig. 1). Insufficient quality of the 3D surface model was defined by the presence of low spatial resolution, motion or imaging artifacts, or incomplete or inaccurate segmentation in the 3D rotational angiography data, all of which could compromise reliable morphological assessment and the accuracy of subsequent hemodynamic simulations. Quality assessment was performed by two experienced researchers—one from the Department of Fluid Dynamics and Technical Flows and one neurosurgeon—ensuring both technical and clinical perspectives. Only models deemed compatible with semi-automatic postprocessing and CFD pipeline requirements were included in the final analysis. The Ethics Committee of the Medical Faculty of the Otto-von-Guericke University reviewed and approved the analysis of this retrospective data, confirming that all procedures were conducted as part of standard clinical care.

### Data acquisition

The clinical data for this study were retrospectively obtained through a comprehensive review of patient records, including detailed medical histories, medication profiles, and pre-existing diagnostic imaging. Epidemiological parameters, such as age at diagnosis, sex, comorbidities, and modifiable risk factors, including nicotine abuse, alcohol consumption, and obesity, were systematically evaluated. To investigate the natural history and clinical management of SIA, detailed data were extracted on the rupture status of the SIA, from diagnostic imaging, on aneurysm-specific risk profiles, and on patient outcomes. The epidemiological, clinical, and aneurysm-specific characteristics of the here analyzed SIAs are summarized in Table 1.

**Table 1:** Epidemiological and clinical characteristics as well as aneurysm specific information of unruptured (*n* = 101) and ruptured (*n* = 40) sias.

	Unruptured (*n* = 101)	Ruptured (*n* = 40)	*p*-value
Epidemiological data			
Sex	m = 24 (23.8%); f = 77 (76.2%)	m = 12 (30.0%); f = 28 (70.0%)	0.6
Mean age at diagnosis (± σ, years)	54.55 (± 11.13)	52.0 (± 13.48)	0.2
Comorbidities and risk factors			
Arterial hypertension	67 (66.3%)	30 (75%)	0.5
Diabetes mellitus type 2	0 (0%)	0 (0%)	0.4
Hypercholesterolemia	27 (26.7%)	7 (17.5%)	0.5
Nicotine abuse	64 (63.4%)	12 (30.0%)	0.0008
Alcohole abuse	11 (10.9%)	2 (5.0%)	0.1
Obesity	23 (22.8%)	14 (35.0%)	0.2
Aneurysm localization			
Anterior cerebral artery	7 (6.9%)	0 (0%)	0.003
Anterior communicating artery	13 (12.9%)	17 (42.5%)	
Pericallosal artery	3 (3.0%)	1 (2.5%)	
Internal carotid artery	25 (24.8%)	6 (15.0%)	
Middle cerebral artery	39 (38.6%)	10 (25.0%)	
Posterior communicating artery	0 (0%)	1 (2.5%)	
Basilar artery	9 (8.9%)	0 (0%)	
Posterior inferior cerebellar artery	1 (1%)	2 (5.0%)	
Others	1 (1%)	0 (0%)	
Aneurysm multiplicity	91 (90.1%)	12 (30%)	< 0.001

The table presents patient demographics, comorbidities, risk factors, and aneurysm localization, highlighting differences between the two groups.

### Morphological analysis

**Fig. 2:**
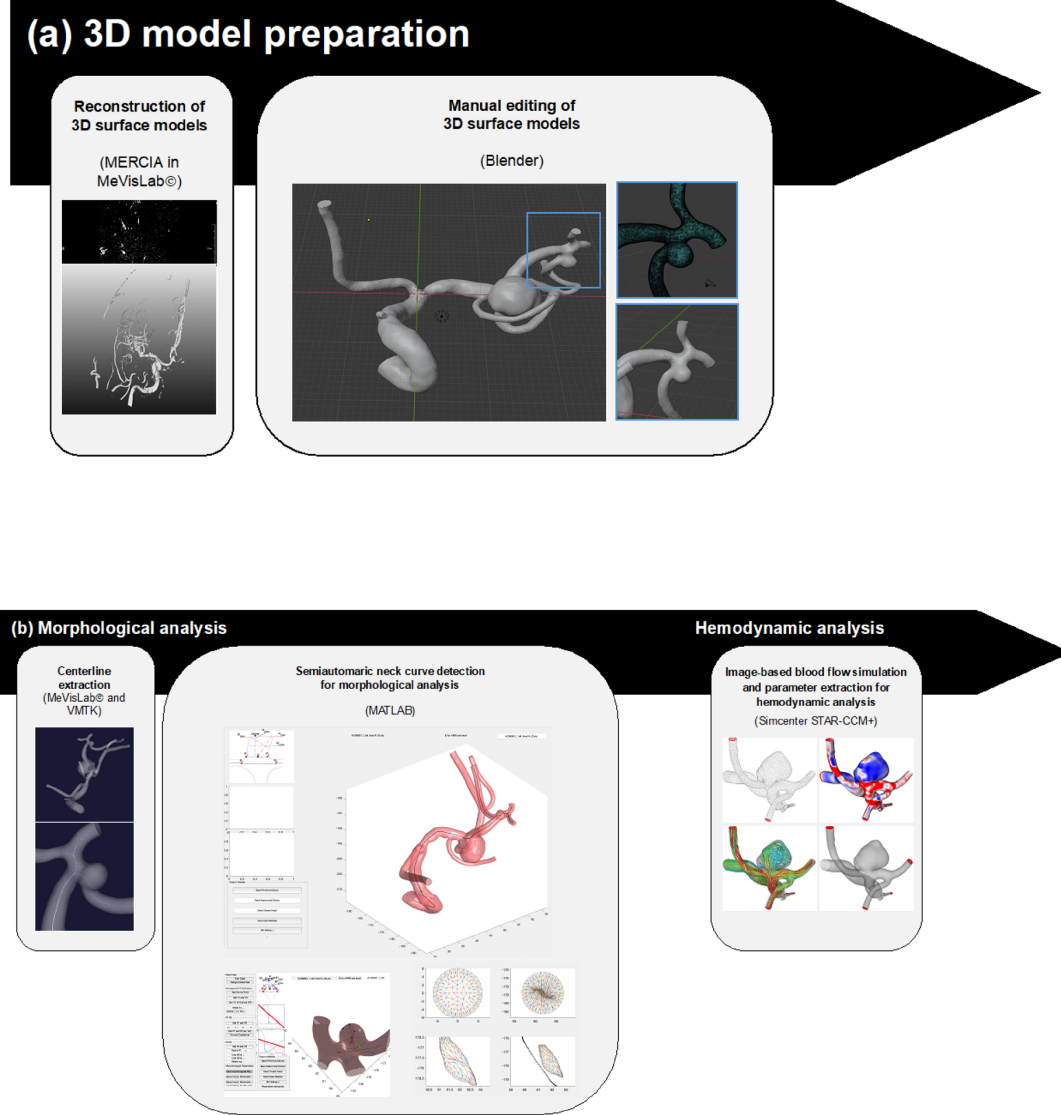
Schematic representation of the stepwise workflow for generating high-fidelity three-dimensional surface models from 3D rotational angiography datasets. The reconstruction was performed using the inhouse software MERCIA within MeVisLab©, followed by manual refinement in Blender to remove artifacts, adjust inlets and outlets, and smooth the mesh. The vascular centerline was extracted using the Vascular Modeling Toolkit (VMTK), and further processing in MATLAB enabled semi-automatic detection of the aneurysm neck curve.

**Fig. 3:**
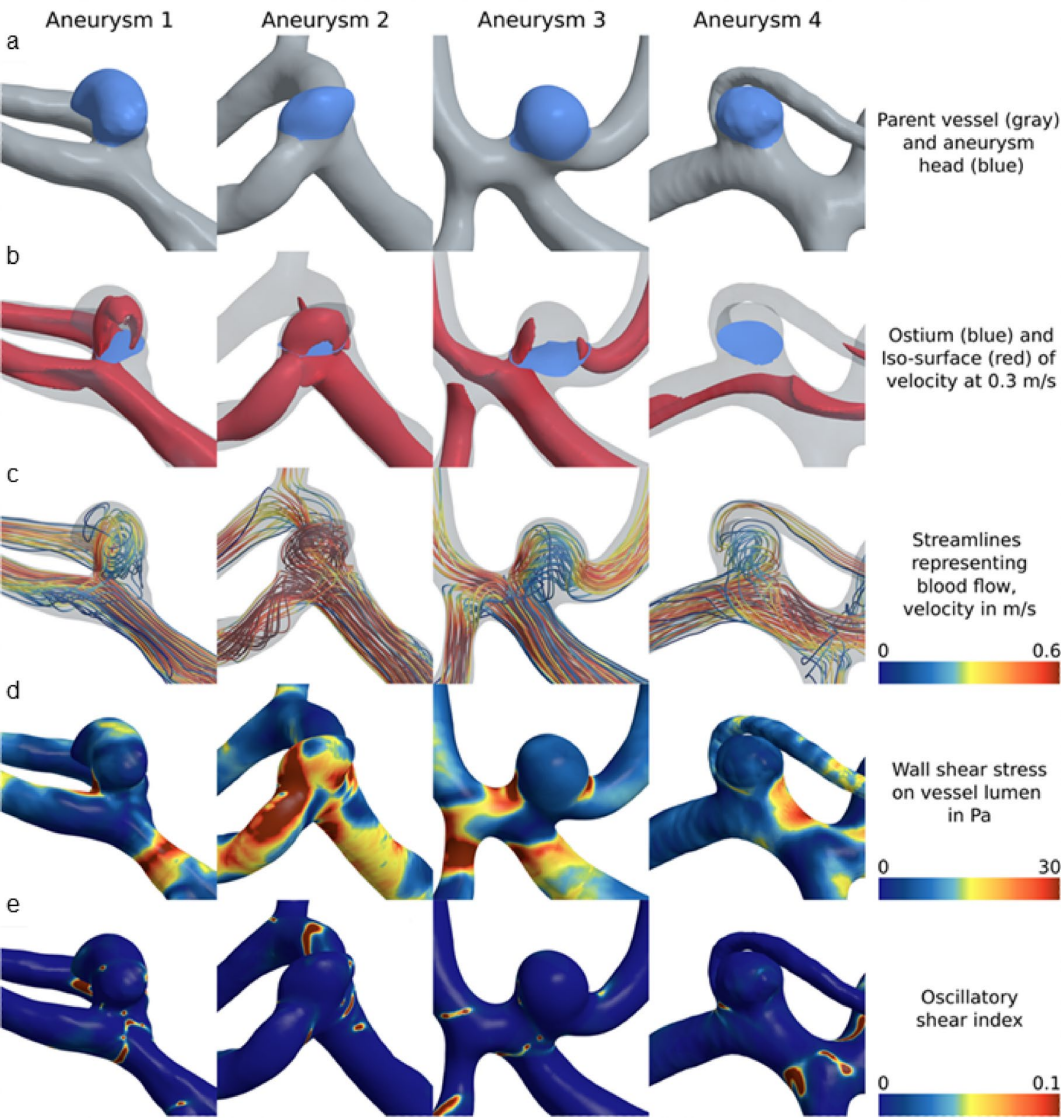
Four representative aneurysms from the study cohort are presented, illustrating morphological and hemodynamic characteristics: (**a**) aneurysm location (blue) at the parent vessel; (**b**) the ostium delineating the aneurysm lumen from the parent vessel (blue) along with the velocity iso-surface; (**c**) streamlines of the temporally averaged flow field color-coded by local blood velocity; (**d**) temporally averaged wall shear stress induced by frictional forces of blood flow; and (**e**) the oscillatory shear index, which describes directional changes in shear forces throughout the cardiac cycle. Aneurysms 1 and 2 are ruptured, whereas Aneurysms 3 and 4 are unruptured. Images were screenshoted from Blender 4.4 (https://www.blender.org), from the inhouse software MERCIA within MeVisLab© (https://www.mevislab.de/de/), the VMTK 1.4.0 (http://www.vmtk.org), the inhouse software for semi-automatic detection of the aneurysm neck curve implemented in MATLAB R2022a (https://www.mathworks.com/products/matlab.html?s_tid=hp_ff_p_matlab) and 2024 Simcenter STAR-CCM+ (https://plm.sw.siemens.com/en-US/simcenter/fluids-thermal-simulation/star-ccm/).

Three-dimensional surface models were reconstructed using 3D rotational angiography datasets and the inhouse software MERCIA, implemented in MeVisLab© (MeVis Medical Solutions AG). The segmentation was carefully refined through manual editing in Blender 2.93.4 (The Blender Foundation, Amsterdam, Netherlands) to ensure the removal of any artifacts and improve overall accuracy. Additionally, the peripheral regions were trimmed, and the inlets and outlets were adjusted to align approximately perpendicular to the vessel centerline. Lastly, the mesh was manually smoothed using the sculpting workspace in Blender (Fig. 2a)^32^. Figure 3a illustrates the surface of four representative SIAs. The centerline was extracted using the Vascular Modeling Toolkit (VMTK) version 1.4.0^33^. Further processing of the centerline was performed in MATLAB R2021a (The MathWorks Inc., Natick, MA, USA), where a specialized algorithm was applied for the semi-automatic detection of the aneurysm neck curve (Fig. 2b)^34^. This approach facilitated the precise automated extraction of 23 key morphological parameters, which are presented in Table 2.

**Table 2:** Comparison of 23 semiautomatically extracted morphological parameters between unruptured (*n* = 101) and ruptured (*n* = 40) sias.

Morphological parameters	Unit	Unruptured (*n* = 101)	Ruptured (*n* = 40)	*p*-value
Maximum diameter	mm	3.73 (± 0.82)	4.11 (± 0.76)	0.01
Maximum height	mm	2.53 (± 0.77)	2.91 (± 0.75)	0.009
Maximum height 2	mm	2.53 (± 0.77)	2.91 (± 0.75)	0.009
Perpendicular height	mm	2.15(± 0.8)	2.27 (± 0.74)	0.4
Perpendicular height 2	mm	2.92 (± 0.81)	2.59 (± 0.75)	0.05
Perpendicular maximum width	mm	3.12 (± 0.76)	3.23 (± 0.69)	0.07
Parallel maximum width	mm	3.33 (± 0.78)	3.59 (± 0.76)	0.4
Average neck diameter	mm	2.79 (± 0.67)	2.81 (± 0.66)	0.8
Maximum neck diameter	mm	3.15 (± 0.73)	3.21 (± 0.76)	0.7
Aneurysm area	mm^2^	22.12 (± 11.07)	25.11 (± 9.75)	0.1
Aneurysm volume	mm^3^	12.73 (± 8.88)	14.18(± 7.6)	0.4
Ostium area 1	mm^2^	6.88 (± 3.04)	7.06(± 3.2)	0.8
Ostium area 2	mm^2^	6.35(± 2.83)	6.35(± 2.78)	1
Aspect ratio 1	–	0.76 (± 0.35)	0.85(± 0.3)	0.2
Aspect ratio 2	–	0.86(± 0.39)	0.96 (± 0.35)	0.1
Convex hull volume	mm^3^	14.26(± 9.53)	16.24 (± 8.33)	0.3
Convex hull surface	mm^2^	29.41 (± 13.08)	32.82 (± 11.46)	0.2
Ellipticity index	–	0.28 (± 0.04)	0.28 (± 0.02)	0.5
Non-sphericity index	–	0.1 (± 0.06)	0.13(± 0.06)	0.01
Undulation index	–	0.13(± 0.06)	0.13 (± 0.07)	0.6
Alpha	degree	64.32 (± 17.55)	72.59 (± 18.68)	0.5
Beta	degree	56.06(± 17.86)	55.37(± 14.0)	0.06
Gamma	degree	59.62 (± 21.60)	52.04 (± 19.3)	0.06

The table presents the mean values and standard deviations (± σ) for each of the analyzed morphological parameters^34^.

### Hemodynamic analysis

The three-dimensional surface models of the lumen geometry were used for highly resolved, time-dependent blood flow simulations. Computational fluid dynamics computations based on the software Simcenter STAR-CCM + 17.06 (Siemens PLM Software Inc., Plano, TX, USA) were conducted within a semi-automatic workflow developed in accordance with the guidelines of Berg et al. (Fig. 2b)^17^. Blood was modeled as incompressible fluid (constant density of 1056 kg/m^3^) with non-Newtonian viscosity behavior (Carreau-Yasuda model parameters taken from Robertson et al.^35^) assuming laminar flow conditions. The choice of boundary conditions has a significant influence on the accuracy of aneurysm hemodynamics. In particular, the inflow boundary condition can lead to considerable deviations^36,37^. As no subject-specific flow waveforms are available, and given the inherently variable nature of such waveforms, a representative inflow curve^38^ is employed and scaled to the local vessel diameter. A representative inflow curve was taken by Cebral et al.^38^ and scaled to the local vessel diameter. At the outlets, an area-weighted outflow splitting technique was applied to distribute the flow appropriately^39^. This approach yields results comparable to those of more complex methods^36,40^. Vessel walls were modeled as rigid. A time step of 0.5 ms was employed. Two cardiac cycles were simulated to ensure accurate and stable flow patterns. The first cardiac cycle served as an initialization phase and the second was used for data acquisition and subsequent analysis. 25 hemodynamic parameters^25,41^ were calculated to characterize the patient-specific blood flow, see Table 3. In addition, Fig. 3 illustrates underlying hemodynamic data for four representative SIAs.

**Table 3:** Comparison of 25 hemodynamic parameters between unruptured (*n* = 101) and ruptured (*n* = 40) sias.

Hemodynamic parameters	Unit	Unruptured (*n* = 101)	Ruptured (*n* = 40)	*p*-value
Maximum WSS	Pa	71.55 (± 62.39)	85.23 (± 53.47)	0.2
Mean WSS	Pa	8.00 (± 6.82)	10.09 (± 7.65)	0.05
Minimum WSS	Pa	3.47E−01 (± 0.71)	2.71E−01 (± 0.37)	0.5
Normalized WSS	–	7.41E−01 (± 0.49)	7.34E−01 (± 0.46)	0.9
Mean WSS of parent artery	Pa	11.89 (± 10.74)	15.59 (± 11.19)	0.04
Flow rate of parent artery	Kg/m^3^	2.63E−03 (± 1.57E−03)	2.99E−03 (± 1.69E−03)	0.2
Neck inflow rate	Kg/m^3^	6.45E−04 (± 5.42E−04)	8.70E−04 (± 6.28E−04)	0.03
Inflow concentration index	–	6.73E−01 (± 0.51)	7.12E−01 (± 0.42)	0.7
Maximum oscillatory shear index	–	3.88E−01 (± 0.12)	4.06E−01 (± 0.09)	0.3
Mean oscillatory shear index	–	1.69E−02 (± 0.02)	1.74E−02 (± 0.02)	0.9
Maximum velocity	m/s	6.33E−01 (± 0.32)	7.38E−01 (± 0.29)	0.03
Mean velocity	m/s	2.21E−01 (± 0.14)	2.66E−01 (± 0.15)	0.06
Pulsatility index of velocity	–	1.65 (± 0.38)	1.55 (± 0.27)	0.08
Pulsatility index of neck inflow rate	–	1.38 (± 0.27)	1.31 (± 0.20)	0.03
Pulsatility index of WSS	–	2.38 (± 0.42)	2.29 (± 0.31)	0.1
Kinetic energy	µJ	5.49E−01 (± 8.64E−01)	8.51E−01 (± 9.59E−01)	0.06
Kinetic energy of parent artery	µJ	2.13E−00 (± 1.97E−00)	2.84E−00 (± 2.28E−00)	0.01
Kinetic energy ratio	–	3.46E−01 (± 0.45)	3.80E−01 (± 0.34)	0.9
High shear area	–	1.35E−01 (± 0.16)	1.38E−01 (± 0.17)	0.8
Low shear area 1*	–	5.13E−01 (± 0.3)	5.05E−01 (± 0.3)	0.7
Low shear area 2*	–	1.52E−01 (± 0.25)	1.20E−01 (± 0.21)	0.6
Low shear area 3*	–	7.74E−02 (± 0.17)	9.95E−02 (± 0.21)	0.4
Shear concentration index	–	3.94 (± 3.57)	3.12 (± 2.06)	0.2
Low shear index	–	2.28E−01 (± 0.25)	2.30E−01 (± 0.28)	0.7
High shear index	–	7.25E−02 (± 0.13)	7.36E−02 (± 0.13)	0.7

The table presents the mean values and standard deviations (± σ) for each of the analyzed hemodynamic parameters. *Low shear area follows three different definitions: (1) aneurysm area with WSS less than one standard deviation below the mean WSS of the parent artery divided by the total aneurysm area, (2) aneurysm area with WSS less 1.5 pa divided by the total aneurysm area and (3) aneurysm area with WSS less 10% of the mean WSS of the parent artery divided by the total aneurysm area.

### Statistical analysis

The dataset was pseudo-randomly divided into a training/validation set and a test set, using sklearn.model_selection. train_test_split() function, and maintaining a size ratio of 2:1. The clinical and demographic parameters were assessed for equal distribution across the two sets and no significant differences were observed. The training and validation set was used for model development, while the test set was assigned exclusively for the final evaluation of the model’s performance on unseen data.

To address non-normal distributions, the Yeo-Johnson transformation^42^—an extension to the perhaps better known Box-Cox transformation, but capable of handling negative numbers – was applied to the morphological and hemodynamic variables. To mitigate multicollinearity and reduce dimensionality, complete-linkage agglomerative clustering was performed to group highly correlated variables, with the clustering threshold set at Pearson’s r2 > 0.25, i.e. |r| > 0.5. Vessel parameters were excluded from this analysis. Within each cluster, simple logistic regression was conducted using cluster members as independent variables (predictors) and rupture status as the dependent variable (outcome). The cluster member with the lowest p-value was selected as the representative for the cluster. Only representatives with *p* < 0.05 were retained for further analysis.

Multiple logistic regression with forward selection and five-fold cross-validation was employed to determine the optimal predictor combination, maximizing McFadden’s pseudo-R^2^. McFadden’s pseudo-R2^43^ is a proper scoring rule based on the log-likelihood of observing the data, given the model parameters, and defined analogously to the better-known original R2, which is used in linear regression. In contrast to metrics like accuracy, AUC, or F1, which are based on discrete predictions (classes) for the data points, McFadden’s pseudo-R2 works with continuous, real-valued likelihoods. The process started with the most significant predictor from the previous step, sequentially adding variables that improved the pseudo-R^2^. The final model’s performance was evaluated on the test set. A similar forward selection process was applied to demographic and clinical variables, including sex, age, arterial hypertension, diabetes, hypercholesterolemia, smoking, alcohol consumption, and obesity. In addition to logistic regression, a selection of machine learning classifiers, including AdaBoost and support vector machines, was applied to assess model robustness. Hyperparameters were optimized through cross-validated grid search to ensure fair comparison across models and configurations.

All statistical computations were carried out using Python, specifically the scipy, scikit-learn, and statsmodels libraries.

## Results

### Demographics

The study cohort comprised 141 patients, of whom 101 (71.6%) had unruptured SIAs and 40 (28.4%) had ruptured SIAs. Women were predominant in both groups, accounting for 76.2% of patients with unruptured SIAs and 70.0% of those with ruptured SIAs. The mean age at diagnosis was slightly higher in the unruptured group (54.55 ± 11.13 years) compared to the ruptured group (52.0 ± 13.48 years).

Aneurysm localization varied markedly between the two groups. In the unruptured group, the middle cerebral artery (MCA) was the most common site (38.6%), followed by the internal carotid artery (24.8%). In contrast, ruptured aneurysms were predominantly located in the anterior communicating artery (ACOM, 42.5%), while MCA aneurysms accounted for only 25.0%. Aneurysm multiplicity was significantly more frequent among patients with unruptured SIAs (90.1%) compared to those with ruptured SIAs (30.0%).

### Predictive modeling

**Fig. 4:**
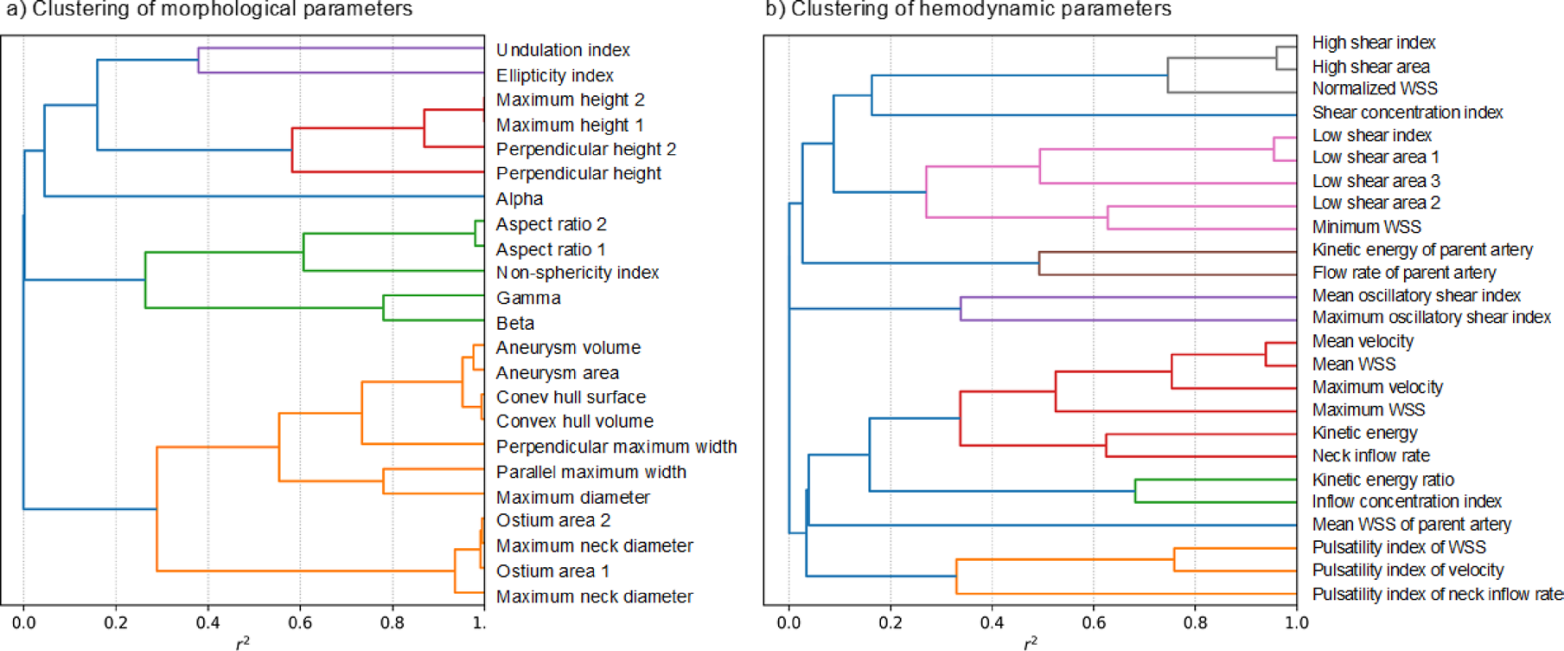
Clustering of morphological (**a**) and hemodynamic (**b**) parameters to avoid multicollinearity in the regression analysis. Highly correlated variables were grouped into clusters, and for each cluster, the variable with the smallest and significant *p*-value in the univariate regression was selected as the representative. The clustering was performed based on the squared Pearson correlation coefficient (r^2^), using a cut-off of r^2^ > 0.25.

Predictive models were developed exclusively on the training/validation dataset, while the test set was used for evaluation. The clusters of related morphological and hemodynamic parameters, obtained through agglomerative clustering, are shown in Fig. 4. The cluster representatives which in simple logistic regression were shown to be significant predictors of the rupture status (*p* < 0.05) are listed in Table 4. A more lenient selection, using *p* < 0.1 as the inclusion criterion, included two additional cluster representatives, the Pulsatility Index for Neck Inflow rate (*p* = 0.08) and Shear Concentration Index (*p* = 0.09), but their inclusion didn’t change the subsequent results. Due to the wide variety of possible aneurysm locations and high imbalance in their presence, in combination with a relatively small data set (“curse of dimensionality”), we couldn’t investigate the predictive power of the location on the rupture.

**Table 4:** Cluster representatives identified through simple logistic regression, where each cluster member was used as an independent variable (predictor) and rupture status as the dependent variable (outcome).

Cluster representative	Parameter class	*p*-value
Maximum height 1	Morphological (Fig. 4a)	0.011
Maximum diameter	Morphological (Fig. 4a)	0.016
Non-sphericity index	Morphological (Fig. 4a)	0.049
Neck inflow rate	Hemodynamic (Fig. 4b)	0.016

The variable with the lowest *p*-value in each cluster was selected as the representative, and only those with *p* < 0.05 were retained for further analysis. Representatives of each cluster, selected based on simple logistic regression, are shown alongside their corresponding p-values. The cluster representatives are chroma-coded, as in Fig. 4.

**Fig. 5:**
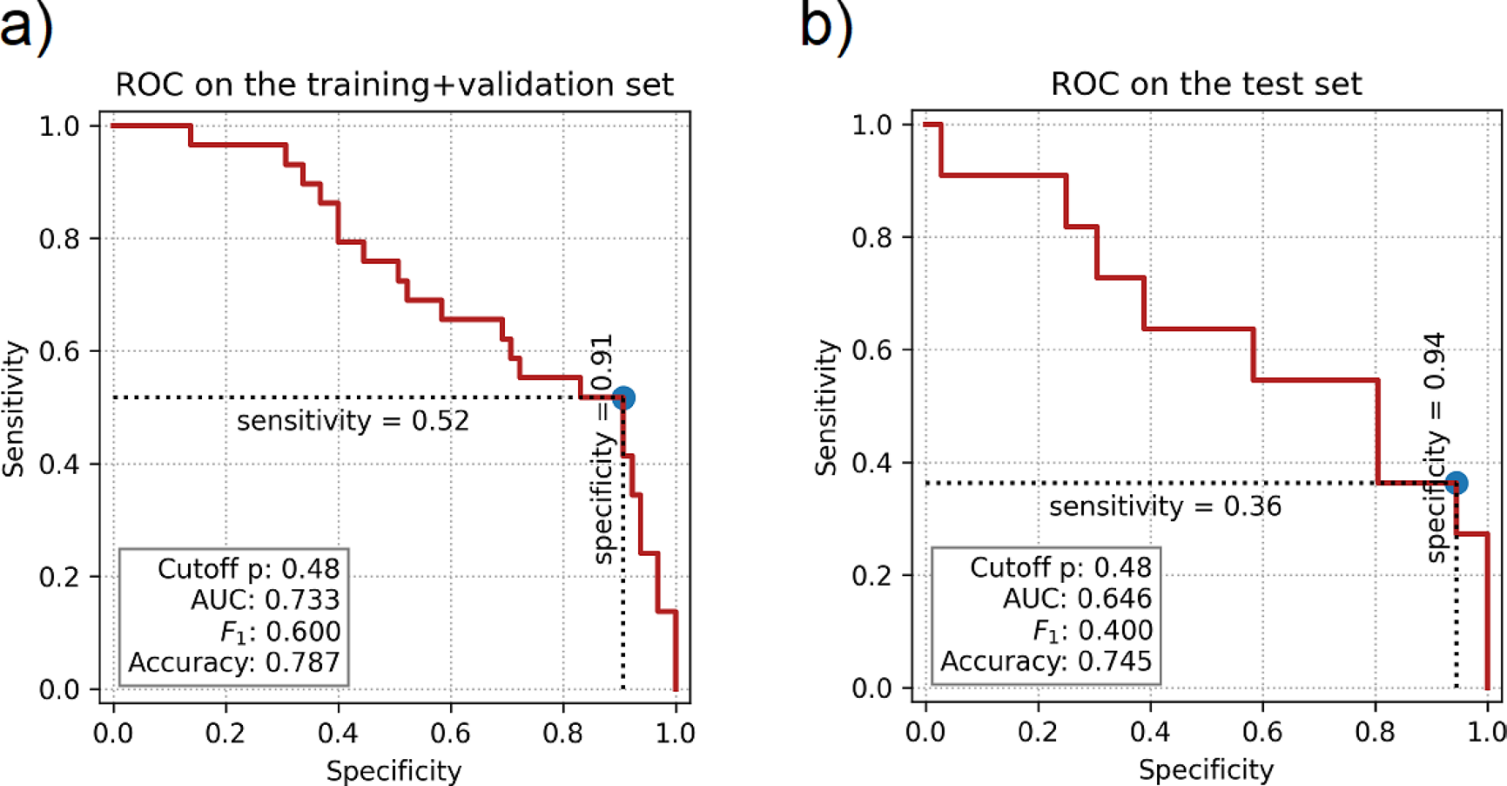
Presentation of the results of the binary classifier based on logistic regression for the simplified model using neck inflow rate and non-sphericity index, applied to the training and validation dataset (**a**) and the test dataset (**b**), with respective accuracies of 0.787 and 0.745.

In further analysis, forward selection based on improving pseudo-R^2^applied to morphological and hemodynamic variables, produced a model with predictors including the maximum height, the Neck inflow rate, and the Non-sphericity index. The latter two showed to be significant predictors, while the maximum height was not. This model achieved a pseudo-R^2^ of 0.142 on the training and validation set but only 0.032 on the test set. For comparison, a simpler model using only the Neck inflow rate and the Non-sphericity index as the predictors yielded the same pseudo-R^2^ on the training and validation set and a slightly better value of 0.034 on the test set. A binary classifier based on the logistic regression achieves an accuracy of 0.745 on the test set, which is lower than the fraction of unruptured aneurysm in the set (0.766) (Fig. 5). These findings suggest that morphological and hemodynamic parameters are not reliable predictors of rupture in small aneurysms. The strong performance of the complex model on the training and validation set likely reflects overfitting, underscoring the importance of validating models on an independent test set.

For clinical variables, none emerged as significant predictors of rupture. Obesity exhibited a weak trend on the training and validation set (*p* = 0.095, pseudo-R^2^ = 0.024), but its performance on the test set dropped to a pseudo-R^2^ of − 0.047, confirming that obesity is not a reliable predictor of SIA rupture.

The prediction performance of various machine learning classifiers trained on the three key predictors—Maximum Height, Neck Inflow Rate, and Non-sphericity Index—is summarized in Table 5. The results demonstrate that none of the classifiers achieved reliably high predictive performance, highlighting the challenge of rupture risk prediction in SIA.

**Table 5:** Performance of different machine learning classifiers on the independent test set using the three key predictors (Maximum height, neck inflow rate, and Non-sphericity Index). Shown are the values for accuracy, precision, sensitivity, specificity, and F1-score.

Classifier	Accuracy	Precision	Sensitivity	Specificity	F1-score
AdaBoost classifier	0.631	0.339	0.359	0.359	0.347
Support vector machine	0.752	0.556	0.154	0.154	0.229
K-nearest neighbors	0.702	0.398	0.231	0.231	0.287
Stochastic gradient descent	0.695	0.265	0.205	0.205	0.224

## Discussion

The management of SIAs remains a clinical challenge, as their low estimated rupture risk must be weighed against the significant morbidity and mortality associated with aneurysmal SAH. This study builds on previous investigations, identifying increased rupture risks in patients with SIAs who are younger than 50 years, have aneurysm diameters ≥ 4 mm, hypertension, or multiple aneurysms^8,14^. The distribution of unruptured (71.6%) and ruptured (28.4%) SIAs in this study is consistent with previous findings^44,45^. The predominance of female patients (76.2% unruptured, 70.0% ruptured) also aligns with earlier reports^46–48^. Aneurysm localization differed between groups, with MCA being most common in unruptured cases (38.6%), while ruptured aneurysms were predominantly found in the ACOM (42.5%). This supports prior studies identifying ACOM aneurysms as the most rupture-prone^49–51^ likely due to their unique hemodynamic conditions. Notably, the rate of aneurysm multiplicity was substantially higher in the unruptured group (90%) compared to the ruptured group (30%). This discrepancy likely reflects a selection bias, as many unruptured aneurysms were identified in patients undergoing treatment for another aneurysm. This limitation is common in retrospective aneurysm datasets.

Recent evidence underscores the pivotal role of morphological and hemodynamic factors in rupture risk assessment for SIAs. Morphological predictors, including size ratio, aspect ratio, bifurcation location, aneurysm irregularity, and hemodynamic parameters such as WSS, have been shown to outperform traditional demographic predictors^15^. This paradigm shift highlights the importance of integrating detailed morphological and hemodynamic analyses into clinical decision-making, offering a more robust and personalized approach to risk stratification and management. The dual-pathway hypothesis proposed for aneurysm progression suggests a link between low WSS and high oscillatory shear index and inflammatory-cell-mediated remodeling, often observed in larger, atherosclerotic aneurysms^31^. Conversely, high WSS and positive WSS gradients might be associated with mural-cell-mediated remodeling, a mechanism prevalent in small, thin-walled aneurysms^31^. This hypothesis suggest that smaller aneurysms may follow distinct biological pathways compared to their larger counterparts, necessitating a tailored approach in rupture risk stratification.

Our analysis of SIAs initially identified three morphological and one hemodynamic parameter as cluster representatives. Through a forward selection process, maximum aneurysm height, Neck inflow rate, and Non-sphericity index emerged as predictors of aneurysm rupture. However, only the Neck inflow rate and the Non-sphericity index demonstrated statistical significance. Despite this, neither the complete model incorporating all predictors nor the simplified model relying solely on the Neck inflow rate and the Non-sphericity index provided reliable performance. While the full model showed marginally acceptable fit on the training set, it performed poorly on the test set. Similarly, the simplified model also failed to generalize, reinforcing concerns about overfitting. Contrary to findings from previous studies^8,15–17^ our cohort analysis revealed that none of the investigated morphological or hemodynamic parameters, including the maximum aneurysm height, achieved sufficient predictive accuracy for rupture status. This finding underscores the complexity of rupture risk assessment in SIA and suggests that traditional predictors may not be universally applicable across cohorts. We can only partially address the dual-pathway hypothesis in our study. Since our analysis focused exclusively on SIAs without comparison to larger aneurysms, establishing a definitive link is challenging. Nevertheless, our findings indicate that neither high nor low WSS contributed significantly to the rupture of SIAs within our cohort.

In numerous previous studies, arterial hypertension has been identified as a significant risk factor for the rupture of SIAs^8,12,14^. Since our analysis of the morphological and hemodynamic characteristics of SIAs did not yield conclusive results, we subsequently investigated established comorbidities and risk factors in patients with SIAs. This included age, sex, arterial hypertension, diabetes, hypercholesterolemia, smoking, alcohol consumption, and obesity. However, these factors similarly failed to reliably predict rupture risk, highlighting the need for a more nuanced approach that incorporates multiple factors beyond the morphological and clinical domains to improve predictive modeling for SIA rupture. The comparable performance of different classifiers points to the complexity of rupture risk as a key limiting factor, rather than the modeling technique itself.

This study has several limitations that should be acknowledged. First, as a retrospective analysis, there is an inherent bias in the data, particularly for unruptured aneurysms. Many of these aneurysms may have undergone treatment during follow-up, leaving the natural progression of the disease unknown. This limits the ability to fully understand the natural history of SIAs and their rupture risk. Second, the patient cohort analyzed in this study was relatively limited in size, which may impact the generalizability of the findings and the statistical power of the predictive models. Importantly, for ruptured aneurysms, the imaging data used for analysis were acquired after the rupture event. As rupture may alter aneurysm morphology, this introduces a potential confounder, as the extracted geometries and derived hemodynamic parameters may not accurately represent pre-rupture conditions. Methodological limitations include potential variability in morphological reconstructions due to imaging quality and manual refinements. Hemodynamic modeling relies on assumptions such as rigid vessel walls and idealized inflow conditions, which may not fully reflect in vivo dynamics.

To address these limitations, future studies should focus on the prospective enrollment of patients to provide a more accurate understanding of the natural history of SIAs. Furthermore, the analysis could be enhanced by employing patient-specific boundary conditions and focusing on hemodynamic parameters that are less sensitive to systemic flow variability. Additionally, establishing a comparative analysis between small and large aneurysms could offer valuable insights into the mechanistic differences in rupture risk and enhance the development of tailored predictive models. The application of AI could help overcome some limitations by improving segmentation accuracy, automating feature selection, and enhancing predictive modeling for rupture risk assessment. Such approaches could improve the clinical management of SIA and support more individualized treatment decisions.

## Conclusion

This study underscores the challenges in predicting rupture risk for SIAs. While the Neck inflow rate and the Non-sphericity index were the only significant predictor among morphological and hemodynamic parameters, the size, the simplified model nor the full model demonstrated reliable performance on independent test data. Clinical variables such as age, sex, and hypertension also failed to predict rupture risk reliably. The consistency of results across multiple classification algorithms further reinforces that the observed limitations are unlikely to be method-dependent. These findings suggest that traditional predictors alone are insufficient for accurate risk stratification in SIAs. Future research should focus on prospective studies with larger datasets, integrating multimodal approaches and comparative analyses between small and large aneurysms to refine predictive models and improve clinical decision-making.

## Data Availability

The data supporting the findings of this study are not openly available due to reasons of sensitivity and are available from the corresponding author upon reasonable request.

## Abbreviations

IA: Intracranial aneurysm
SAH: Subarachnoid hemorrhage
SIA: Small intracranial aneurysm
WSS: Wall shear stress
CFD: Computational fluid dynamics
MCA: Middle cerebral artery
ACOM: Anterior communicating artery
